# Investigating effects of soil chemicals on density of small mammal bioindicators using spatial capture-recapture models

**DOI:** 10.1371/journal.pone.0238870

**Published:** 2020-09-17

**Authors:** Shannon M. Gaukler, Sean M. Murphy, Jesse T. Berryhill, Brent E. Thompson, Benjamin J. Sutter, Charles D. Hathcock

**Affiliations:** 1 Environmental Stewardship Group, Los Alamos National Laboratory, Los Alamos, New Mexico, United States of America; 2 Department of Forestry and Natural Resources, University of Kentucky, Lexington, Kentucky, United States of America; 3 Infrastructure Program Office, Los Alamos National Laboratory, Los Alamos, New Mexico, United States of America; Sichuan University, CHINA

## Abstract

Monitoring the ecological impacts of environmental pollution and the effectiveness of remediation efforts requires identifying relationships between contaminants and the disruption of biological processes in populations, communities, or ecosystems. Wildlife are useful bioindicators, but traditional comparative experimental approaches rely on a staunch and typically unverifiable assumption that, in the absence of contaminants, reference and contaminated sites would support the same densities of bioindicators, thereby inferring direct causation from indirect data. We demonstrate the utility of spatial capture-recapture (SCR) models for overcoming these issues, testing if community density of common small mammal bioindicators was directly influenced by soil chemical concentrations. By modeling density as an inhomogeneous Poisson point process, we found evidence for an inverse spatial relationship between *Peromyscus* density and soil mercury concentrations, but not other chemicals, such as polychlorinated biphenyls, at a site formerly occupied by a nuclear reactor. Although the coefficient point estimate supported *Peromyscus* density being lower where mercury concentrations were higher (β = –0.44), the 95% confidence interval overlapped zero, suggesting no effect was also compatible with our data. Estimated density from the most parsimonious model (2.88 mice/ha; 95% CI = 1.63–5.08), which did not support a density-chemical relationship, was within the range of reported densities for *Peromyscus* that did not inhabit contaminated sites elsewhere. Environmental pollution remains a global threat to biodiversity and ecosystem and human health, and our study provides an illustrative example of the utility of SCR models for investigating the effects that chemicals may have on wildlife bioindicator populations and communities.

## Introduction

Wildlife populations and communities have played critical roles as bioindicators of environmental contamination for decades. Numerous species have been used to evaluate the presence of heavy metals and persistent organic pollutants in ecosystems, examine the associated physiological effects of bioaccumulation, and determine the effectiveness of environmental remediation efforts [1–3]. Given the growing human population and the prevalence of environmental pollution as a global threat to biodiversity, ecosystems, and human health [4–7], wildlife likely will continue to be used as bioindicators into the foreseeable future [8, 9]. General consensus exists regarding the criteria that make a particular wildlife species useful as a bioindicator [2, 10, 11], and studies at the individual level of the molecular, cellular, and physiological effects of pollutants on wildlife are prevalent [12]. Despite promising achievements and revealing findings, the impacts of environmental pollutants on wildlife at higher levels of biological organization remain poorly understood. Indeed, a major challenge to developing reliable predictions about the ecological ramifications of pollution and evaluating the effectiveness of remediation efforts is causally linking pollutants to disruption of natural biological processes in populations, communities, and ecosystems [12–14].

A commonly employed experimental design for investigating population- or community-level effects of environmental contaminants is the comparative impacted versus reference approach, or treatment versus control [15]. For instance, no appreciable differences were found in white-footed mice (*Peromyscus leucopus*) population sizes or densities between reference sites and sites contaminated with heavy metals or polychlorinated biphenyls (PCBs), leading researchers to conclude that contaminant levels had little to no adverse population-level effects [16–18]. In contrast, discrepancies were detected in population sizes or densities of American mink (*Neovison vison*), North American river otter (*Lontra canadensis*), and Eurasian otter (*Lutra lutra*) between reference sites and PCBs-contaminated sites, which, in some cases, led to the invocation of immediate changes to environmental remediation practices [19–21]. However, such comparative experimental designs rely on a very strong but typically unverifiable assumption that the impacted and reference sites would support the same population sizes or densities had pollution never occurred [22]. In addition, it is common that this type of approach does not account for differential animal detectability among sites, generally provides facile mono-explanations that oversimplify ecological complexities, and typically fails to sufficiently establish direct causality [12, 13, 22, 23]. As Linzey and Grant [16] and Phelps and McBee [18] noted, observed differences in population demographics among impacted and reference sites may instead be the result of variation in other exogenous factors that are often not evaluated; for example, quantity and quality of available food resources, habitat heterogeneity, predation pressure, disease type and prevalence, or competition with heterospecifics [24, 25]. Thus, across the fields of ecotoxicology and pollution biomonitoring, researchers have called for the adoption or development of analytical methods that facilitate explicit identification and quantification of relationships between pollutants and populations, communities, or ecosystems [12–14].

Individual heterogeneity in demographic parameters is influenced by animals’ interactions with the environment, which ultimately affects population dynamics [26–28]. For example, high levels of persistent organic pollutants may reduce survival or reproductive rates of individual seabirds, which can perturb overall population growth [29, 30]. Population density is an ecological demographic parameter that represents the culmination of important vital rates, reflects the environmental conditions that exert effects on populations, and is therefore a robust metric for quantifying population-environment relationships [24, 25]. Conventional methods for estimating density of wildlife populations rely on first estimating population size (abundance), typically via non-spatial capture-recapture models [31], and then applying estimated population size *ad hoc* to a notional ‘effective sampling area’ [32]. However, because spatial information about captures and recaptures of animals relative to trap locations is not incorporated in conventional capture-recapture models, the effective sampling area is unknown and must be arbitrarily delineated; and individual heterogeneity in detection probabilities that arises due to the varying proximity of animal activity (home range) centers to traps is difficult to accommodate in conventional models [32, 33]. Consequently, densities that are derived from population sizes estimated by conventional capture-recapture models tend to be positively biased, sometimes by ≥200% [34–36].

Relatively recently developed spatial capture-recapture (SCR) models surmount the aforementioned issues by including a state model for the spatial distribution of animal activity centers across a defined geographical area and a distance-dependent detection model in which detection probability declines with increasing distance between an animal’s activity center and a trap [32, 33, 37, 38]. Whereas population size is the estimated parameter of interest in conventional capture-recapture models, SCR models directly estimate density. Unbiased estimates of density can be produced by SCR models [32, 33, 37], which easily accommodate detection data from a multitude of sampling methods (e.g., live-capture, camera-trapping, transect and area searches, bioacoustics, etc.), and have been successfully applied to a wide array of taxa, from amphibians to songbirds to large mammals [34, 36, 39].

Modeling animal density using a homogeneous Poisson point process state model, or that animal activity centers are spatially distributed at random (density is constant across space), is a common approach with SCR models. A unique feature of SCR models is that hypotheses about spatial variation in animal density due to population-environment relationships can be formally tested by replacing the homogenous Poisson point process model with an inhomogeneous Poisson point process model, which allows animal density to spatially vary with values of environmental covariates [32, 33, 35]. For example, using SCR inhomogeneous Poisson point process models, important relationships between American black bear (*Ursus americanus*) density and forest cover, human development, elevation, and hydrology have been revealed and quantified [40–42]. Effectively, SCR inhomogeneous Poisson point process models provide a straightforward approach for testing whether animal density is directly related to measurable environmental conditions.

Here, we demonstrate the utility of SCR models for investigating and quantifying whether density of small mammal bioindicators is directly influenced by local concentrations of chemicals in soil; specifically, inorganic elements (i.e., metals) and organic chemicals, such as PCBs. *Peromyscus* spp. (deer mice) are effective bioindicators of environmental contamination in terrestrial environments, because they have a wide-spread distribution, generally occur in large numbers, are easy to survey and capture, and have high exposure to potential contaminants in vegetation and soil due to their omnivorous diet and burrowing behavior [43, 44]. Furthermore, high concentrations of some chemicals, such as PCBs, have been linked to developmental issues, abnormal liver, spleen, and adrenal functions, and reduced survival and reproductive rates of *Peromyscus*, all of which could result in population reductions [45, 46]. Therefore, we hypothesized that density of a *Peromyscus* community would be inversely related to concentrations of chemicals in soil, or more specifically, that density would be lower where chemical concentrations were higher.

## Methods

### Study area

Los Alamos National Laboratory (LANL) was established in northern New Mexico, USA, in 1943 as part of the Manhattan Project, primarily to design and build atomic weapons. LANL is situated on a series of mesas of the Pajarito Plateau, which are subdivided by multiple east-west oriented canyons. Our study occurred in Los Alamos Canyon, where some of the earliest nuclear operations in the United States occurred. Five experimental nuclear reactors were active in Los Alamos Canyon during 1944–1992, and demolition of the final reactor occurred in 2002 [47]. As a result of reactor operations, multiple inorganic and organic chemicals are of environmental monitoring interest [48–50].

The width of Los Alamos Canyon within our study area is approximately 400 m, and elevation ranges from 2,100 m at the canyon floor to 2,200 m at the canyon rim. Habitat on the north-facing canyon slope and in the canyon bottom is mixed conifer; and piñon pine (*Pinus edulis*) and one-seed juniper (*Juniperus monosperma*) habitat comprise the south-facing canyon slope [51]. The climate is semi-arid with an average annual precipitation of 48 cm and mean annual temperature of 9.1 C° [52].

### Small mammal trapping

**Fig 1:**
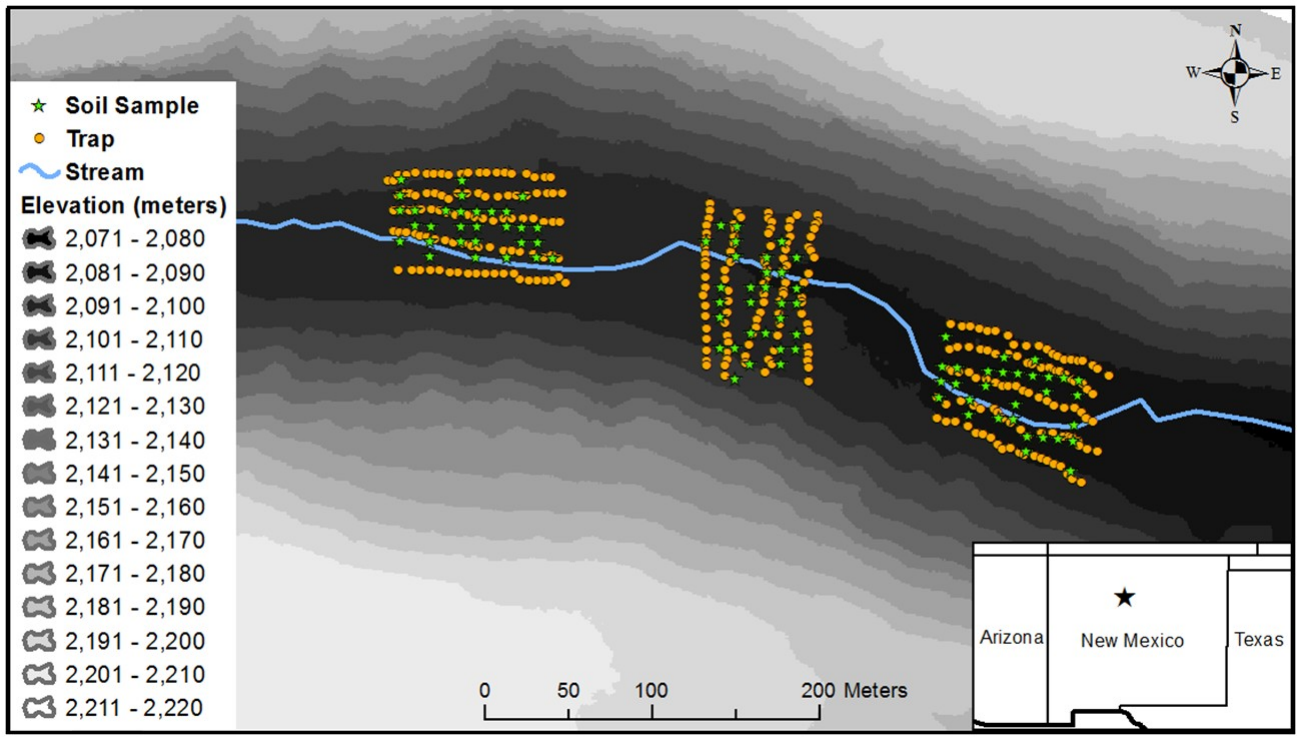
The locations of three trapping grids in Los Alamos Canyon, Los Alamos National Laboratory, New Mexico, USA, relative to the stream and gradient of the canyon’s elevation. Figure was created using ArcGIS 10.4.1.

We conducted a five-day capture-mark-recapture survey during April 2017 by live-capturing small mammals using folding Sherman traps (7.62 × 8.89 × 22.86 cm; H.B. Sherman Traps, Tallahassee, Florida). We placed one hundred traps in five rows of 20 traps in each of three trapping grids, for a total of 300 traps; spacing between traps within grids averaged 4.55 m and spacing between the centers of grids averaged ~170 m (Fig 1). The western-most trapping grid was located on the former footprint of one reactor, and the other two grids were placed east and downstream. We baited traps with a mixture of sweet feed and peanut butter each night and checked all traps the following morning. We identified the species and sex of all captured animals and recorded additional morphological information. We marked each captured individual with a uniquely numbered ear tag when first captured, which enabled individual identification in subsequent capture occasions. We released all captured individuals near their respective capture locations. All capture and handling methods were approved by an Institutional Animal Care and Use Committee (protocol #16–30), followed standardized guidelines for humane capture and handling of wildlife [53], and occurred under a New Mexico Department of Game and Fish scientific collection permit (#2864).

### Soil collection and chemical concentrations

We collected soil samples from each of the three trapping grids for chemical analysis. We first divided each grid equally into six subgrids, and within each subgrid, we collected one composite soil sample from five randomly selected locations. Soil samples were collected with a 10-cm-diameter soil ring that was driven 5 cm into the ground with a hammer. We homogenized all five composite samples from a subgrid in a sealed plastic bag, which we then aliquoted into a sample container for chemical analysis. We collected a total of 18 composite samples from 18 subgrids (six from each of the three grids; Fig 1).

Soil samples were analyzed for inorganic elements at Australian Laboratory Services (Fort Collins, Colorado), and were analyzed for PCBs and dioxin and furan congeners at Cape Fear Analytical, LLC (Wilmington, North Carolina; see S1 Appendix for more details). Radionuclides were not assessed, because they were not identified as chemicals of potential ecological concern. The World Health Organization developed toxic equivalency factors (TEFs) for tetrachlorinatedibenzodioxin-2,3,7,8 (TCDD)-like compounds, which can be used to determine the relative potency or toxic equivalents (TEQs) of dioxin-like compounds [54]. We calculated TEQs for all aryl hydrocarbon-binding PCB, dioxin, and furan congeners by multiplying each congener by its respective mammalian-specific TEF [54], which we then added together for a total TEQ for each soil sample.

### Statistical analyses

#### Soil chemistry

We assigned all chemicals that were nondetects to a concentration equal to the detection limit (i.e., the laboratory’s detection or reporting limits, based on standardized reporting). A nondetect is a chemical concentration that is somewhere between zero and the analytical laboratory’s reporting limit but is too small to accurately quantify [55]. Reporting nondetects at the detection limit is preferred over other methods, such as reporting at half the detection limit, substituting with zeros, or disregarding them entirely, provided statistical analyses are used that can accommodate censored data [55]. For datasets containing nondetect values (i.e., censored), we made comparisons among groups using a Gehan-Wilcoxon test [56]. For datasets that did not contain nondetect values, we used a nonparametric Kruskal-Wallis One-Way ANOVA on Ranks test. If support existed for substantial differences in the concentrations among grids (*P* < 0.05), we applied a post hoc Dunn’s test with a Bonferroni correction for multiple pairwise comparisons.

#### Kriging

Kriging is a commonly used geostatistical modeling technique to probabilistically interpolate or predict values across space from a set of observed values. We applied empirical Bayesian kriging, implemented in the ArcGIS Pro 2.2 Geostatistical Wizard (ESRI, Redlands, CA), to predict chemical concentration values at locations across the study area where samples were not collected, based on the spatial locations of observed concentrations. Empirical Bayesian kriging tends to produce more accurate predictions from small datasets compared with other kriging approaches [57, 58]. We only applied kriging to chemicals that had concentrations that were supported as being different among the three grids (*P* < 0.05), which suggested that sufficient variation existed to quantify potential density-chemical relationships. Many dioxin and furan congeners met this criterion, so we used TEQs as surrogates.

Prior to kriging, we assessed each chemical for outliers and normality using an iterative Grubbs test and a Shapiro-Wilk test, respectively [59, 60]. We log-empirical transformed non-normal data, because soil chemical concentrations cannot be negative; values were back-transformed for estimating prediction surfaces. We removed outliers from the analysis to improve prediction accuracy, but included them in the final prediction surfaces [61]. We applied all semi-variograms that are available under empirical Bayesian kriging, which are spatial autocorrelation models that predict the relationship of concentrations as a function of distance and direction. We selected the best semi-variogram for each chemical via cross-validation, based on the following goodness-of-fit measures: mean error (ME), mean standardized error (MSDE), mean standard error (MSE), root-mean-square error (RMSE), and root-mean-square standardized error (RMSSDE) [58, 62–64].

#### Spatial capture-recapture

We fit single-session closed population SCR models by maximizing the full likelihood in the R statistical software package *secr* v3.1.7 [65, 66] to estimate *Peromyscus* density and density-chemical relationships. The live-traps that we used were single-catch, which introduces dependence among traps and trap-specific competition among individuals [32, 67]. A likelihood estimator for single-catch traps has not been developed yet (but see Distiller and Borchers [68]), but Efford et al. [67] found that modeling single-catch traps as multi-catch can produce unbiased estimates of density if trap saturation is <86%. Therefore, after calculating trap saturation, or the average proportion of traps that were occupied at the end of each occasion, we modeled traps as multi-catch via a multinomial observation model [32, 67, 69]. Recaptures of multiple individuals occurred in traps that were located on >1 sampling grid (i.e., cross-grid captures), effectively constituting a pseudo-clustered sampling design; therefore, we collapsed the grid-specific capture histories into a single study area capture history with five occasions [41, 70, 71].

We fit all models under the common assumption that the probability of capturing an individual decreased with increasing distance between a trap and the animal’s activity center according to a half-normal curve [32, 37]. This detection function has two parameters that are estimated, the probability of capture at the activity center of an individual (*g*_*0*_) and the spatial scale of detection (σ), the latter of which reflects how rapidly capture probability declines with distance from a trap [32, 33]. A default assumption of SCR models is that animal home ranges are approximately circular, but this is often violated in natural populations due to a myriad of factors, such as territoriality, heterogeneous distribution of resources, or movement-restricting landscape features [72, 73]. If home ranges are elongated in a particular direction (e.g., because of landscape features that restrict movement, such as canyons or rivers) and traps are deployed primarily along the major axis of animal movement such that the trapping array aligns with the directionality of home ranges, estimated density may be severely biased under the circularity assumption [72]. Our trapping grids were collectively oriented along the direction of Los Alamos Canyon (the *x*-axis), with minimal trap coverage along the *y*-axis, and exploratory analyses suggested that animal movement may have been predominantly aligned with the canyon. Therefore, we applied an anisotropic transformation to the detection function, which has been shown to mitigate density estimate bias in such scenarios [72, 74]. We implemented the anisotropic transformation using the R package *geoR* [75, 76], specified the anisotropy angle parameter (Φ_A_) as 105° (1.83 radians) based on the orientation of the canyon, and estimated the anisotropy ratio parameter (Φ_R_) via maximum likelihood [74].

To test the importance of potential sources of variation in detection function parameters, we initially used the default SCR homogeneous Poisson point process model to describe the spatial distribution of animal activity centers across the state space (*S*), or area of integration. We defined *S* by buffering each trap by the recommended ~3× σ estimated from the most parsimonious model to ensure that all activity centers of individuals with a non-negligible probability of capture were included [32]. We specified a fine-resolution mask point spacing of 10 m for *S* to conform to the recommended <1× σ that is needed to eliminate bias that can be caused by discretization of *S* [32, 77]. We then fit models with the following effects modeled on *g*_*0*_ and/or σ, per the recommendations of Gerber and Parmenter [78] for SCR analysis of small mammal detection data. Because traps were baited, we modeled a trap-specific learned behavioral response (bk) on g_*0*_ [74, 79]. Male *Peromyscus* sometimes have larger home ranges than females [80], which could influence detection and movement rates; therefore, we fit models with sex as a grouping covariate on *g*_*0*_ and/or σ [81–83]. Additionally, because three species of *Peromyscus* were captured during our study, which might have differential space use and detection rates among them [80], we also fit models with species as a grouping covariate on g_*0*_ and/or σ [83]. To investigate if individual heterogeneity in detection or space use existed that was not accounted for by sex or species, we fit models with latent two-class finite mixtures (π) on g_*0*_ and/or σ [84, 85].

To investigate density-chemical relationships, we modeled the spatial distribution of individual activity centers across *S* as an inhomogeneous Poisson point process. Specifically, we modeled *Peromyscus* density as a log-linear function of the concentrations of manganese, mercury, PCBs, and TEQ values in soil [32]. Inhomogeneous Poisson point process SCR models are computationally demanding; therefore, we fit these models with only the effects on detection function parameters for which 95% confidence intervals of coefficient estimates did not overlap zero (for bk and π) or overlap among sexes or species in the aforementioned homogeneous Poisson point process models. We used Akaike’s Information Criterion corrected for small sample size (AIC_*c*_) for model selection, wherein we conservatively considered models ≤4 ΔAIC_*c*_ units from the top model as competing [86, 87]. However, we also followed the additional recommendations by Arnold [87] for direct *a priori* hypothesis testing, whereby we report the results of models that represent hypothesis-related manipulations of key model parameters (i.e., density varying as a function of chemical concentrations) even if uninformative parameters were present.

## Results

### Small mammal trapping

We captured 31 individual *Peromyscus* (20 males: 11 females) 78 total times in 49 separate traps (16.3% of all traps) across 1,500 trap-nights; 30 captures were males and 48 captures were females. Three species were captured and the number of captures varied among them, with individual *P*. *boylii* (*n* = 17), *P*. *maniculatus* (*n* = 4), and *P*. *truei* (*n* = 10) being captured 43, 12, and 23 times, respectively. With the species combined, the total number of captures within an occasion ranged from 13 to 18, and 47 total recaptures were obtained, with 44 of those being spatial recaptures (i.e., recaptured in a different trap than the initial capture). The average number of recaptures per individual was 1.52 (range: 0–5) and six individuals were captured on >1 trapping grid. Trap saturation across all occasions was 5.20%, which was sufficiently below the threshold required to mitigate bias in SCR density estimates from modeling single-catch traps as multi-catch [67, 68].

### Soil chemistry

Substantial differences among the three trapping grids for concentrations of multiple chemicals were not supported (*P* > 0.05; A1 Table in S1 Appendix). However, manganese concentrations were higher in soil collected from the lower grid compared with soil from the upper grid (*P* = 0.04). In contrast, mercury concentrations were higher in soil collected from the upper grid compared with soil from the middle and lower grids (*P* = 0.02). Similarly, PCBs concentrations were higher in soil samples collected from the upper grid compared with soil samples from the middle and lower grids (*P* < 0.001). Most dioxin and furan congener concentrations in soil samples varied among grids, with soil in the upper grid generally having higher concentrations than soil from the middle and lower grids (A1 and A2 Tables in S1 Appendix); therefore, we used TEQs as a surrogate. TEQs were higher in soil samples from the upper grid compared with soil samples collected from the middle and lower grids (*P* < 0.01).

### Kriging

**Fig 2:**
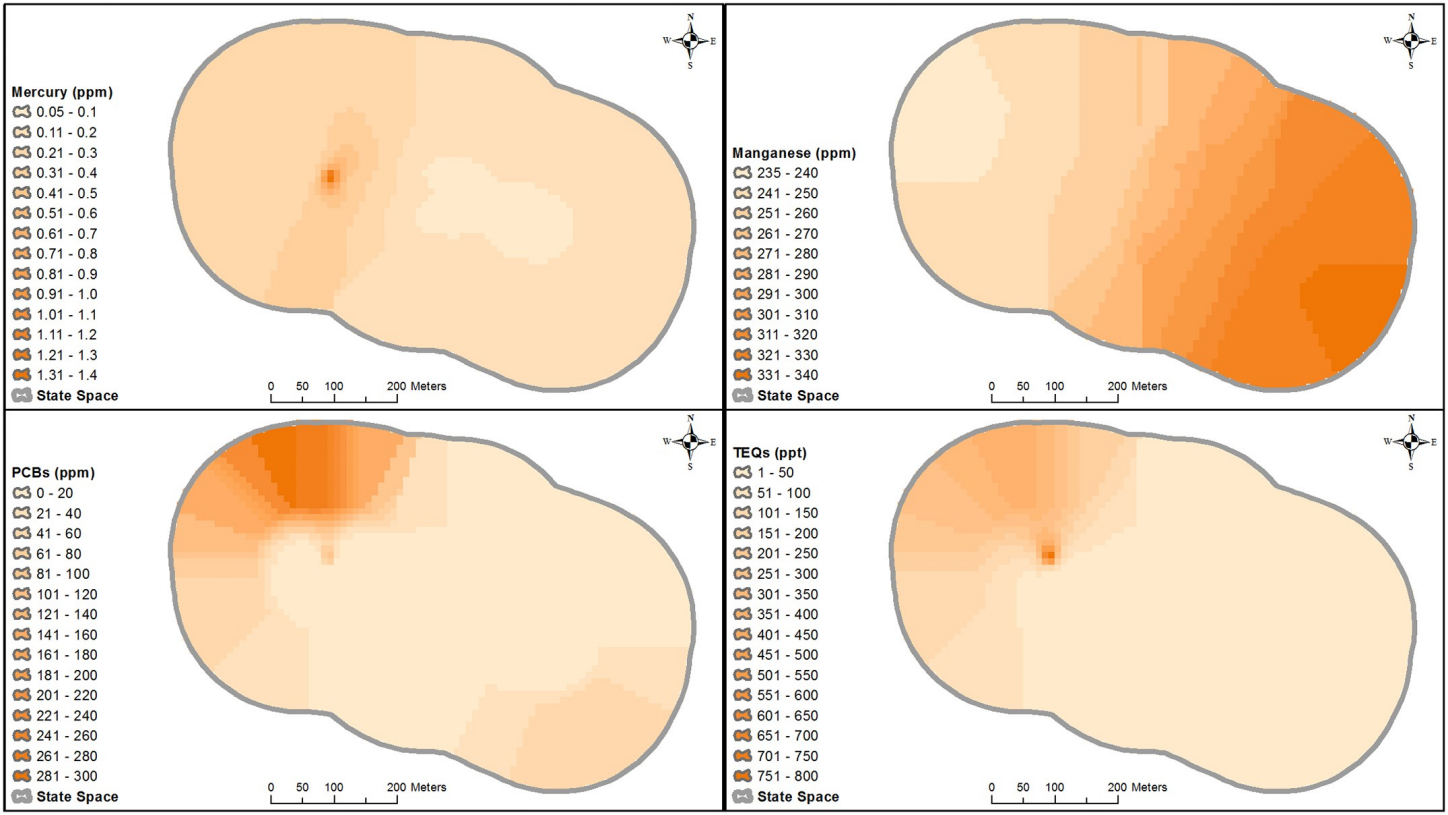
Spatial distributions of mercury, manganese, PCBs, and TEQs concentrations (ppm = parts per million, ppt = parts per trillion) in soil of Los Alamos Canyon, produced from interpolation using empirical Bayesian kriging models. Figure was created using ArcGIS 10.4.1.

Manganese contained no outliers (Iterative Grubbs α = 0.01) and followed a normal distribution (Shapiro-Wilk W = 0.96, *P* = 0.61). Mercury, PCBs, and TEQs all contained two outliers (Iterative Grubbs α = 0.01) and were non-normally distributed even after the removal of the outliers (Shapiro-Wilk W = 0.66, *P* < 0.0001; W = 0.78, *P* < 0.01; W = 0.71, *P* < 0.001, respectively). Based on cross-validation using the goodness-of-fit criteria, we selected the exponential semi-variogram for both PCBs and TEQs, a whittle semi-variogram for mercury, and a power semi-variogram for manganese (B1 Table in S2 Appendix). Prediction accuracy for all four chemicals was acceptable, as general agreement existed between MSE and RMSE for each. Nominal bias was present in the estimated variability (as measured by MSDE and RMSSDE), but slight underestimation of the variation in mercury and TEQs may have been present (RMSSDE = 0.83 and 0.82, respectively). The final predicted surfaces of chemical concentrations are shown in Fig 2.

### Spatial capture-recapture

Anisotropic transformation of the detection function was supported over the default isotropic detection function (ΔAIC_*c*_ = 2.1; B2 Table in S2 Appendix), indicating that *Peromyscus* home ranges were generally elongated and aligned with the directionality of the canyon and orientation of trapping grids (Φ_R_ = 1.7, 95% CI = 1.2–2.5; B1 Fig in S2 Appendix). A positive trap-specific behavioral response (bk) on g_*0*_ was supported (β_bk_ = 2.0, 95% CI = 0.95–3.0), but sex effects on g_*0*_ and σ were not well supported (β_*g0*(sex)_ = -0.58, 95% CI = -1.6–0.42; β_σ(sex)_ = -0.16, 95% CI = -0.64–0.33). Although some support existed for *P*. *maniculatus* and *P*. *truei* having slightly smaller σ than *P*. *boylii* (β_σ(PEMA)_ = -0.84, 95% CI = -1.3–-0.09; β_σ(PETR)_ = -0.57, 95% CI = -1.1–-0.06), 95% confidence intervals overlapped among species and negligible support existed for *g*_*0*_ differing among species (β_*g0*(PEMA)_ = 1.5, 95% CI = -0.04–3.1; β_*g0*(PETR)_ = 0.47, 95% CI = -0.72–1.7). Two-class finite mixtures on *g*_*0*_ and σ were strongly supported (β_*g0*(π)_ = 2.5, 95% CI = 1.1–3.9; β_σ(π)_ = -1.6, 95% CI = -1.1–-2.1), indicating that non-spatial individual heterogeneity in detection and space use existed that was not explained by sex or species. Therefore, we retained a trap-specific behavioral response on *g*_*0*_ and two-class mixtures on *g*_*0*_ and/or σ in all inhomogeneous Poisson point process models, but excluded species and sex effects given the nominal support for either.

The top model had an AIC_*c*_ weight of 0.58, whereas all five competing models (≤4 ΔAIC_*c*_) had low AIC_*c*_ weights of 0.08–0.09 (Table 1). The two highest ranked models included density as a homogeneous Poisson point process, whereas the four other competing models included density as an inhomogeneous Poisson point process in which density varied as a function of chemical concentrations in soil. Some support, although limited, existed for *Peromyscus* density spatially varying across the study area with mercury concentrations (β_Hg_ = -0.44, 95% CI = -4.1–3.2), but density varying with concentrations of manganese, PCBs, or TEQs was not supported (β_Mn_ = -0.001, 95% CI = -0.01–0.01; β_PCB_ = -0.002, 95% CI = -0.01–0.01; β_TEQ_ = 0.001, 95% CI = -0.004–0.005). The most parsimonious top model included homogeneous density and estimated that 29% of *Peromyscus* had low *g*_*0*_ (0.04, 95% CI = 0.01–0.13) and large σ (71 m, 95% CI = 40–128), whereas 71% had high *g*_*0*_ (0.31, 95% CI = 0.10–0.65) and small σ (14 m, 95% CI = 8.9–24). Given those σ estimates, we buffered all traps by 200 m, because the probability of detection at this distance was effectively zero (B1 Fig in S2 Appendix), which resulted in a 36-ha area of integration (*S*; state space). Estimated community density across *S* was 2.9 animals/ha (95% CI = 1.6–5.1), which corresponded to a total of 104 individual *Peromyscus* (95% CI = 58–183). That estimate indicated that we captured and marked 30% (95% CI = 17–53%) of the *Peromyscus* community within the study area.

**Table 1:** Model selection of competing (≤4 ΔAIC_*c*_) spatial capture-recapture models that estimated *Peromyscus* density (*D*) in Los Alamos Canyon. We fit models that included a trap-specific behavioral response (bk) on the probability of detection at the activity center of an individual (*g*_*0*_), and allowed *g*_*0*_ to vary between sexes (Sex), among species (Species), between latent two-class mixtures (π), or to be constant (~1). We also allowed the spatial scale of detection (σ) to vary by sex, species, π, or to be constant. Density was modeled as a homogeneous (~1) or inhomogeneous Poisson point process, the latter of which allowed the spatial distribution of animal activity centers to vary with concentrations of manganese (Mn), mercury (Hg), PCBs, or TEQs in soil. The full model selection list can be viewed in B2 Table in S2 Appendix.

Model	K^a^	AIC_*c*_^b^	ΔAIC_*c*_^c^	ω_*i*_^d^	logLik^e^	Deviance^f^
*D*(~1) *g*_*0*_(~bk + π) σ(~π) Φ(~1)	8	860.5	0.0	0.58	–419.0	838.0
*D*(~1) *g*_*0*_(~bk) σ(~π) Φ(~1)	7	864.2	3.7	0.09	–422.7	845.3
*D*(~PCB) *g*_*0*_(~bk + π) σ(~π) Φ(~1)	9	864.4	3.8	0.09	–419.0	837.8
*D*(~Mn) *g*_*0*_(~bk + π) σ(~π) Φ(~1)	9	864.5	3.9	0.08	–419.0	837.9
*D*(~Hg) *g*_*0*_(~bk + π) σ(~π) Φ(~1)	9	864.5	4.0	0.08	–419.0	837.9
*D*(~TEQ) *g*_*0*_(~bk + π) σ(~π) Φ(~1)	9	864.5	4.0	0.08	–419.0	837.9

^a^ Number of model parameters.

^b^ Akaike’s Information Criterion corrected for small sample size.

^c^ Relative difference between AIC_*c*_ of model and the highest ranked model.

^d^ Model weight.

^e^ log-likelihood of model.

^f^ Model deviance = –2 × (log-likelihood).

## Discussion

Density is a more ecologically informative demographic parameter than abundance [24, 25, 32], which prior studies that evaluated the impacts of environmental contaminants on small mammal bioindicators recognized [16, 18, 21, 88]. Most of those studies used conventional capture-recapture models, however, and consequently may have derived inaccurate estimates of density [34–37]. Furthermore, because of the limitations of conventional capture-recapture models, prior studies were forced to use a comparative experimental approach to infer potential effects of environmental contaminants on density from indirect data, without the means to explicitly test underlying assumptions. Spatial capture-recapture models are collectively a type of spatial point process model, so one of their primary advantages is that density can be modeled as a function of spatial covariates to directly test population- or community-environment relationships [32, 33, 35].

Sutherland et al. [21] provided the first example of the effectiveness of SCR models for estimating density of a wildlife bioindicator in the context of environmental contamination. Although the findings from that study were revealing, the authors used a traditional comparative experimental approach that was predicated on the untestable assumption that in the absence of PCBs contamination, both study areas would support the same densities of American mink [15, 22]. Those authors did apply SCR inhomogeneous Poisson point process models to investigate if mink density spatially varied with distance from the pollution source, but they did not directly model PCBs concentration on mink density. In contrast, we explicitly modeled *Peromyscus* density as an inhomogeneous Poisson point process to test relationships between density and concentrations of multiple chemicals in soil. Coefficient point estimates for all density-chemical relationships that we analyzed suggested that *Peromyscus* density may have been lower where chemical concentrations in soil were higher. Among the inhomogeneous Poisson point process models that we fit, the most support existed for an inverse relationship between mercury concentrations and density; mercury has been implicated in deleterious effects observed in populations of *Peromyscus* and other wildlife elsewhere [89, 90]. However, we cautiously interpret this density-mercury relationship, because the 95% confidence interval overlapped zero, indicating that no effect was also compatible with the data. Therefore, these results and any associated conclusions must be considered in the context of SCR model assumptions, our model specifications, and our study design, which we discuss below [91, 92].

Accuracy of density estimated by SCR models is often dependent on the capture-recapture survey duration [32, 93]. Most small mammal bioindicators, including *Peromyscus*, are *r*-selected species that have high reproductive and mortality rates and generally short lifespans compared with *K*-selected large mammals [94, 95]. Therefore, short survey periods should be used for *r*-selected species to mitigate bias that can result from violating the demographic closure assumption of single-session capture-recapture models [32, 37]; however, the potentially optimal survey period length for *r*-selected species that can produce density estimates with both high precision and accuracy is approximately 14–30 days [93]. Relative to the life history characteristics of *Peromyscus*, our five-day survey may have been too short to obtain sufficient data for precisely estimating density-chemical relationships. This is supported by the very large estimated coefficient of variation for all density-chemical coefficient estimates (CV > 250%), which was reflected in the wide confidence intervals for the density-mercury relationship. However, considering the estimated number of *Peromyscus* in the study area (*N* = 104 individuals), we likely captured and marked 30% of the community, which is not a small sample size. Furthermore, the number of individuals that we captured, the number of spatial recaptures that we obtained, and our survey period length were similar to most other capture-recapture studies of *Peromyscus*. For example, Linzey and Grant [16] captured an average of 16 individual *Peromyscus* during each of their three-day survey periods, Phelps and McBee [18] captured an average of 34 individual *Peromyscus* during each of their five-day survey periods, and Gerber and Parmenter [78] captured an average of 37 individual *Peromyscus* during each of their five-day survey periods. Nevertheless, recent studies have identified survey durations being overly brief as a widespread and common problem with small mammal capture-recapture studies in general [93, 96–98].

Although the three grids that we collected soil samples from appeared to have covered sufficient variation in the soil concentrations of chemicals, the kriging models relied on concentrations obtained from just 18 composite samples. Compositing samples from multiple subgrids may have diluted or concentrated the overall soil chemical concentrations and thus, might not be the most appropriate collection method if the end goal is to estimate concentrations over a broader spatial extent via kriging. However, compositing soil samples prior to chemical analyses allowed for a greater generalization of chemical loads in the study area via kriging. Whether our small sample size was sufficient for accurately interpolating values across the entire study area is unclear; although, empirical Bayesian kriging models are typically accurate with small datasets [57, 58] and our cross-validation results suggested that prediction accuracy for each chemical was high [58, 62–64]. Additionally, RMSSDE and MSDE values indicated that the kriging models accurately estimated the variance in the predictions, though nominally poorer for mercury and TEQs. Selection of a different semi-variogram creates a unique raster to represent chemical concentrations, and although the differences among semi-variograms are typically minimal [58], selection could potentially influence quantification of density-chemical relationships using SCR models. All semi-variograms for each chemical produced similar rasters of predicted values, and many cross-validation criteria differed only slightly among semi-variograms, suggesting negligible influences of semi-variogram selection.

The specification of our inhomogeneous density SCR models assumed that any relationships between the soil chemicals and the *Peromyscus* community would be linear and direct (i.e., from soil to individuals). The three species that we captured during our study all exhibit prolific burrowing behavior and similar microhabitat selection, so this was a reasonable assumption founded on species ecology [99, 100]; pooling data from multiple *Peromyscus* species is also a very common approach for both environmental contamination and capture-recapture studies [16, 18, 78]. Nevertheless, the influence of environmental chemicals on *Peromyscus* density could also occur through indirect pathways. For example, physiological effects of chemical exposure on individual animals could suppress vital rates, such as survival or reproduction, which consequently influences community abundance and density [45, 46]. Additional indirect pathways through which environmental contaminants could influence *Peromyscus* density include, but are not limited to, perturbation of vegetation growth that degrades the distribution and availability of concealing cover or food resources [101, 102], or altering the density or spatial distribution of heterospecific resource competitors (e.g., shrews [*Soricidae* spp.]; [103]).

Although SCR models account for spatial heterogeneity in detection probabilities, it is vital to also investigate non-spatial sources of variation in detection function parameters (*g*_*0*_ and σ) to improve the accuracy of estimated density [32]. The lack of support for species-specific variation in either *g*_*0*_ or σ strongly indicates that our density estimates were representative of a *Peromyscus* community that was comprised of multiple species with comparable space use and detectability [80]. We also found no evidence that *g*_*0*_ or σ differed between the sexes, though we acknowledge the short survey duration that resulted in a relatively small number of sex-specific spatial recaptures may have contributed to this result [69, 82, 85]. Nevertheless, our estimated community density was similar to other spatially explicit estimates for *Peromyscus* in locales where environmental contamination was not a known issue [78, 96, 98]. Considering the uncertainty depicted by the confidence intervals for the negative density-mercury coefficient estimate, the existing concentrations of mercury in soil likely had a nominal adverse community-level effect on *Peromyscus* in Los Alamos Canyon during our survey period.

## Conclusions

Environmental pollution is a prominent threat to ecosystem and human health that is likely to worsen as human populations increase in size and distribution globally. Robust analytical methods are needed that can identify and quantify relationships between pollutants and populations or communities of wildlife bioindicators to evaluate the ecological consequences of pollution and the effectiveness of remediation efforts. Our study demonstrates how SCR models can be used in a traditional hypothesis-testing framework to evaluate biological and ecological effects of environmental contaminants on animal density and space use. For studies similar to ours that focus on terrestrial small mammal bioindicators, we suggest the following to attempt to improve investigations of the impacts that environmental chemicals in soil may have on populations or communities: 1) extend the duration of capture-recapture surveys to obtain sufficient spatial detection data without violating the demographic closure assumption; 2) increase the spatial intensity of non-composite soil sampling (i.e., number and extent of samples) to reduce uncertainty in kriging predictions; and 3) attempt to include heterospecifics in the capture-recapture survey to account for possible resource/space competition. An optimal research approach may be one that incorporates said recommendations in a comparative experimental design, but that analyzes detection data from each site using SCR models with an inhomogeneous Poisson point process density model to test if bioindicator densities at multiple sites, or in multiple populations or communities, are directly influenced by chemical concentrations.

## Supporting information

S1 Appendix Supplemental tables of results comparing the concentrations of inorganic elements, dioxin congeners, and furan congeners in soil samples collected in Los Alamos Canyon, New Mexico, USA. (DOCX)

S2 Appendix Supplemental tables and figures depicting goodness-of-fit criteria for empirical Bayesian kriging of chemical concentrations, and results from spatial capture-recapture model analysis of *Peromyscus* detection data. (DOCX)

S1 File A zip folder containing all data files necessary for repeating the analyses of soil chemicals and *Peromyscus* spatial capture-recapture. (ZIP)
