# Supplementary material for: Investigating effects of soil chemicals on density of small mammal bioindicators using spatial capture-recapture models
Source: PLoS One. 2020 Sep 17;15(9):e0238870. doi: 10.1371/journal.pone.0238870 (PMC7498087; doi:10.1371/journal.pone.0238870)
Supplement: S1 Appendix — (DOCX) [file pone.0238870.s001.docx]

**Investigating effects of soil chemicals on density of small mammal bioindicators using spatial capture-recapture models**

***PLOS ONE***

**Shannon M. Gaukler, Sean M. Murphy, Jesse T. Berryhill, Brent E. Thompson, Benjamin J. Sutter, and Charles D. Hathcock**

**Corresponding Author Shannon Gaukler email: sgaukler@lanl.gov**

Appendix S1. Supplemental tables of results comparing the concentrations of inorganic elements, dioxin congeners, and furan congeners in soil samples collected in Los Alamos Canyon, New Mexico, USA.

*Additional details for obtaining chemical concentrations*

Antimony, arsenic, cadmium, lead, selenium, silver, and thallium concentrations were measured by inductively coupled plasma mass spectrometry (EPA SW-846 Method 6020A); aluminum, barium, beryllium, chromium, cobalt, copper, iron, manganese, nickel, vanadium, and zinc were measured by inductively coupled plasma atomic emission spectrometry (EPA SW-846 Method 6010B). Mercury was measured by cold-vapor atomic absorption procedure (EPA SW-846 Method 7471A). Soil samples were analyzed for PCB congeners by EPA Method 1668A and for dioxin/furan congeners by EPA SW-846 Method 8290. PCB congeners 156 and 157 co-eluted and were therefore treated as one; the World Health Organization’s toxic equivalency factors are the same for both. In addition to reporting individual congener concentrations, total PCB, dioxin, and furan concentrations were also reported as an additive of all homologs, and when a homolog contained all nondetects, that particular homolog was assigned a zero.

**Table A1** Inorganic element concentrations (mean ± standard deviation) in composite soil samples collected within the study area. Dissimilar capital letters in parentheses indicate support existed for differences among sampling grids (*P* < 0.05). Six composite soil samples were collected from each grid; concentrations are reported in parts per million (ppm)

| Element | Upper Grid | Middle Grid | Lower Grid | *P*-value |
| --- | --- | --- | --- | --- |
| Aluminum | 3885.0 ± 1468.4 | 3688.3 ± 845.0 | 3150.0 ± 726.9 | 0.6 |
| Antimony | 1.0 ±0.1^a^ ND = 6 | 1.0–1.2 ND = 6 | 1.0–1.1 ND = 6 | 1.0 |
| Arsenic | 1.3 ± 0.3 | 1.2 ± 0.4 | 1.1 ± 0.1 | 0.56 |
| Barium | 58.6 ± 23.4 | 66.0 ± 32.5 | 47.5 ± 14.2 | 0.61 |
| Beryllium | 0.5 ± 0.1 | 0.6 ± 0.2 | 0.5 ± 0.1 | 0.52 |
| Cadmium | 0.47–0.51 ND = 6 | 0.49–0.58 ND = 6 | 0.49–0.54 ND = 6 | 1.0 |
| Chromium | 7.0 ± 2.1 | 4.2 ± 1.2 | 6.0 ± 2.9 | 0.17 |
| Cobalt | 1.6 ± 0.8 | 1.3 ± 0.5 | 1.3 ± 0.3 | 0.69 |
| Copper | 7.0 ± 4.7 | 8.4 ± 5.5 | 4.8 ± 1.0 | 0.55 |
| Iron | 9037.0 ± 2120.0 | 7967.0 ± 933.0 | 7190.0 ± 1158.0 | 0.18 |
| Lead | 11.6 ± 3.0 | 11.8 ± 1.4 | 15.7 ± 4.9 | 0.22 |
| Manganese | 240.2 ± 38.7 (A) | 271.8 ± 32.9 (AB) | 322.3 ± 71.9 (B) | 0.04 |
| Mercury | 2.41 ± 5.01 (A) | 0.09 ± 0.13 (B) | 0.05 ± 0.02 (B) | 0.02 |
| Nickel | 4.0 ± 1.6 | 3.4 ± 1.1 | 3.0 ± 0.5 | 0.48 |
| Selenium | 0.6 ± 0.1 | 0.7 ± 0.3 | 0.6 ± 0.3 | 0.71 |
| Silver | 0.5–0.5 ND = 6 | 0.49 ± 0.01^b^ ND = 5 | 0.5–0.5 ND = 6 | 0.37 |
| Thallium | 0.2 ± 0.0 ND = 4 | 0.2 ± 0.0 ND = 5 | 0.2 ± 0.0 ND = 5 | 0.87 |
| Vanadium | 11.7 ± 4.9 | 8.3 ± 2.3 | 7.8 ± 1.9 | 0.22 |
| Zinc | 48.4 ± 14.2 | 45.8 ± 7.8 | 43.7 ± 8.2 | 0.72 |

^a^If all samples were nondetect (ND) for a particular analyte, the range (–) of ND is reported, because the Kaplan-Meir approach does not estimate a mean or standard deviation when all samples are ND.

^b^Nondetect values are sample specific and, therefore, all ND are provided a value. These ND values were used to calculate the mean and standard deviation.

**Table A2** Concentrations (mean ± standard deviation) of the following dioxin congeners in composite soil samples collected within the study area: tetrachlorodibenzodioxin (Tetra), Pentachlorodibenzodioxin (Penta), hexachlorodibenzodioxin (Hexa), heptachlorodibenzodioxin (Hepta), and octachlorodibenzodioxin (Octa). Dissimilar capital letters in parentheses indicate support existed for differences among sampling grids (*P* < 0.05). Six composite soil samples were collected from each grid; concentrations are reported in parts per trillion (ppt)

| Dioxins | Upper Grid | Middle Grid | Lower Grid | *P*-Value |
| --- | --- | --- | --- | --- |
| Tetra [2,3,7,8-] | 0.12 ± 0.05^a^ ND = 5 | 0.09–0.11^b^ ND = 6 | 0.10–0.28 ND = 6 | 0.32 |
| Tetra (Total) | 0.02 ± 0.05 ND = 5 | 0.09 ± 0.13 ND = 4 | 0.08 ± 0.12 ND = 4 | 0.33 |
| Penta [1,2,3,7,8-] | 1.71 ± 1.49 (A) ND = 1 | 0.46–0.48 (B) ND = 6 | 0.46 ± 0.00 (B) ND = 5 | < 0.001 |
| Penta (Total) | 1.77 ± 2.45 ND = 2 | 3.04 ± 3.33 ND = 3 | 0.28 ± 0.43  ND = 4 | 0.11 |
| Hexa [1,2,3,4,7,8-] | 1.39 ± 1.34 ND = 2 | 0.48 ± 0.04 ND = 5 | 0.47 ± 0.02 ND = 5 | 1.0 |
| Hexa [1,2,3,6,7,8-] | 4.45 ± 4.51 (A) ND = 1 | 0.87 ± 0.52 (B) ND = 1 | 0.95 ± 0.55 (B) ND = 1 | < 0.01 |
| Hexa [1,2,3,7,8,9-] | 2.52 ± 2.38 (A) ND = 1 | 0.63 ± 0.20 (B) ND = 3 | 0.83 ± 0.30 (AB) ND = 1 | 0.02 |
| Hexa (Total) | 37.04 ± 40.8 (A) ND = 1 | 6.06 ± 5.27 (B) | 4.91 ± 4.10 (B) | 0.01 |
| Hepta [1,2,3,4,6,7,8-] | 202.6 ± 281.6 (A) | 25.94 ± 20.18 (B) | 23.26 ± 16.56 (B) | 0.01 |
| Hepta (Total) | 472.4 ± 521.8 (A) | 76.52 ± 102.90 (B) | 46.52 ± 34.58 (B) | 0.02 |
| Octa [1,2,3,4,6,7,8,9-] | 1865.0 ± 2623.0 (A) | 223.1 ± 191.0 (B) | 199.1 ± 155.6 (B) | 0.01 |

^a^Nondetect (ND) values are sample-specific and therefore, all ND are provided a value. These ND values were used to calculate the mean and standard deviation.

^b^If all samples were ND for a particular analyte, the range (–) of ND is reported, because the Kaplan-Meir approach does not estimate a mean or standard deviation when all samples are ND.

**Table A3** Concentrations (mean ± standard deviation) of the following furan congeners in composite soil samples collected within the study area: tetrachlorodibenzofuran (Tetra), pentachlorodibenzofuran (Penta), hexachlorodibenzofuran (Hexa), heptachlorodibenzofuran (Hepta), and octachlorodibenzofuran (Octa). Dissimilar capital letters in parentheses indicate support existed for differences among sampling grids (*P* < 0.05). Six composite soil samples were collected from each grid; concentrations are reported in parts per trillion (ppt)

| Furans | Upper Grid | Middle Grid | Lower Grid | *P*-Value |
| --- | --- | --- | --- | --- |
| Tetra [2,3,7,8-] | 247.13 ± 566.62 (A) | 0.34 ± 0.24 (B) ND = 3 | 0.61 ± 0.39 (B) ND = 1 | < 0.001 |
| Tetra (Total) | 1050.00 ± 2520.00 (A) | 1.17 ± 1.48 (B) ND = 1 | 1.21 ± 0.90 (B) | < 0.001 |
| Penta [1,2,3,7,8-] | 68.99 ± 164.62 (A) | 0.46 ± 0.00 (B) ND = 5 | 0.50 ± 0.11 (B) ND = 5 | < 0.001 |
| Penta [2,3,4,7,8-] | 370.72 ± 886.44 (A) | 0.47 ± 0.03 (B) ND = 5 | 0.58 ± 0.17 (B) ND = 3 | < 0.001 |
| Penta (Total) | 2040.00 ± 4830.00 (A) | 2.34 ± 1.70 (B) | 5.44 ± 4.61 (B) | < 0.01 |
| Hexa [1,2,3,4,7,8-] | 358.12 ± 794.85 (A) | 0.75 ± 0.36 (B) ND = 3 | 0.83 ± 0.41 (B) ND = 3 | < 0.001 |
| Hexa [1,2,3,6,7,8-] | 89.54 ± 212.86 (A) | 0.47 ± 0.02 (B) ND = 5 | 0.53 ± 0.10 (B) ND = 3 | < 0.001 |
| Hexa [1,2,3,7,8,9-] | 16.16 ± 34.86  ND = 4 | 0.46–0.48^b^ ND = 6 | 0.46–0.48 ND = 6 | 0.11 |
| Hexa [2,3,4,6,7,8-] | 106.23 ± 249.77 (A) | 0.52 ± 0.07 (B) ND = 3 | 0.67 ± 0.22 (B) ND = 3 | < 0.001 |
| Hexa (Total) | 1322.62 ± 3095.23 (A) | 7.71 ± 0.00 (B) | 8.01 ± 0.00 (B) | 0.01 |
| Hepta [1,2,3,4,6,7,8-] | 221.40 ± 470.16 (A) | 8.12 ± 0.00 (B) | 5.76 ± 0.00 (B) | < 0.02 |
| Hepta [1,2,3,4,7,8,9-] | 67.31 ± 159.57 | 0.65 ± 0.23 ND = 3 | 0.57 ± 0.22  ND = 4 | 1.0 |
| Hepta (Total) | 546.92 ± 1080.0 (A) | 25.65 ± 26.83 (B) | 15.87 ± 11.73 (B) | 0.02 |
| Octa [1,2,3,4,6,7,8,9-] | 592.58 ± 1230.0 (A) | 18.90 ± 18.3 (B) | 12.91 ± 9.94 (B) | 0.01 |

^a^Nondetect (ND) values are sample-specific and therefore, all ND are provided a value. These ND values were used to calculate the mean and standard deviation.

^b^If all samples were ND for a particular analyte, the range (–) of ND is reported, because the Kaplan-Meir approach does not estimate a mean or standard deviation when all samples are ND.
