# Supplementary material for: Investigating effects of soil chemicals on density of small mammal bioindicators using spatial capture-recapture models
Source: PLoS One. 2020 Sep 17;15(9):e0238870. doi: 10.1371/journal.pone.0238870 (PMC7498087; doi:10.1371/journal.pone.0238870)

**Investigating effects of soil chemicals on density of small mammal bioindicators using spatial capture-recapture models**

***PLOS ONE***

**Shannon M. Gaukler, Sean M. Murphy, Jesse T. Berryhill, Brent E. Thompson, Benjamin J. Sutter, and Charles D. Hathcock**

**Corresponding Author Shannon Gaukler email: sgaukler@lanl.gov**

Appendix S2. Supplemental tables and figures depicting goodness-of-fit criteria for empirical Bayesian kriging of chemical concentrations, and results from spatial capture-recapture model analysis of *Peromyscus* detection data.

**Table B1** Goodness-of-fit criteria of selected empirical Bayesian kriging semi-variograms for chemical concentrations in soils collected from Los Alamos Canyon. Criteria included mean error (ME), mean standardized error (MSDE), mean standard error (MSE), root-mean-square error (RMSE), root-mean-square standardized error (RMSSDE). The ranges of concentrations were on different numerical scales among the chemicals evaluated; thus, cross-chemical comparisons of goodness-of-fit criteria were not possible. Therefore, to assess bias, precision, and accuracy of predicted values and variation for each chemical, we relied on the following comparisons among goodness-of-fit statistics: agreement (closeness) between MSE and RMSE, closeness to zero for MSDE, and closeness to one for RMSSDE. All models of the same chemical were cross-validated, and the strongest model was selected following chemical-specific comparisons among the goodness-of-fit criteria to assess prediction accuracy

| Chemical | Fitted Model | ME | MSDE | MSE | RMSSDE | RMSE |
| --- | --- | --- | --- | --- | --- | --- |
| Manganese | Power | 0.25 | 0.006 | 55 | 0.97 | 54 |
| Mercury | Whittle | 0.0005 | 0.001 | 0.14 | 0.83 | 0.11 |
| PCBs | Exponential | 0.004 | 0.004 | 0.12 | 0.99 | 0.07 |
| TEQs | Exponential | 0.00000009 | 0.007 | 0.000007 | 0.82 | 0.000005 |

**Table B2** Spatial capture-recapture model selection for estimating *Peromyscus* density (*D*) in Los Alamos Canyon. We fit models with a multinomial observation model and a half-normal anisotropic detection function (Φ); a null model with the default isotropic detection function is also presented for comparison. We considered a trap-specific behavioral response (bk) on the probability of detection at the activity center of an individual (*g_0_*), which was also allowed to vary between sexes (Sex), among species (Species), between latent two-class mixtures (π), or was constant (~1). We allowed the spatial scale of detection (σ) to vary by sex, species, π, or to be constant. Density was modeled as a homogeneous (~1) or inhomogeneous Poisson point process, the latter of which allowed the spatial distribution of animal activity centers to vary with concentrations of manganese (Mn), mercury (Hg), PCBs, or TEQs in soils

| Model | K^a^ | AIC*_c_*^b^ | ∆AIC*_c_*^c^ | ω*_i_*^d^ | logLik^e^ |
| --- | --- | --- | --- | --- | --- |
| *D*(~1) *g_0_*(~bk + π) σ(~π) Φ(~1) | 8 | 860.53 | 0.00 | 0.58 | –418.99 |
| *D*(~1) *g_0_*(~bk) σ(~π) Φ(~1) | 7 | 864.19 | 3.66 | 0.09 | –422.66 |
| *D*(~PCB) *g_0_*(~bk + π) σ(~π) Φ(~1) | 9 | 864.36 | 3.83 | 0.09 | –418.89 |
| *D*(~Mn) *g_0_*(~bk + π) σ(~π) Φ(~1) | 9 | 864.48 | 3.95 | 0.08 | –418.95 |
| *D*(~Hg) *g_0_*(~bk + π) σ(~π) Φ(~1) | 9 | 864.50 | 3.97 | 0.08 | –418.96 |
| *D*(~TEQ) *g_0_*(~bk + π) σ(~π) Φ(~1) | 9 | 864.52 | 3.99 | 0.08 | –418.97 |
| *D*(~1) *g_0_*(~bk + π) σ(~1) Φ(~1) | 7 | 870.14 | 9.61 | 0.00 | –425.63 |
| *D*(~1) *g_0_*(~bk) σ(~1) Φ(~1) | 5 | 870.86 | 10.33 | 0.00 | –429.23 |
| *D*(~PCB) *g_0_*(~bk) σ(~1) Φ(~1) | 6 | 873.73 | 13.20 | 0.00 | –429.11 |
| *D*(~Mn) *g_0_*(~bk) σ(~1) Φ(~1) | 6 | 873.80 | 13.27 | 0.00 | –429.15 |
| *D*(~TEQ) *g_0_*(~bk) σ(~1) Φ(~1) | 6 | 873.89 | 13.36 | 0.00 | –429.19 |
| *D*(~Hg) *g_0_*(~bk) σ(~1) Φ(~1) | 6 | 873.92 | 13.39 | 0.00 | –429.21 |
| *D*(~1) *g_0_*(~1) σ(~1) Φ(~1) | 4 | 877.85 | 17.32 | 0.00 | –434.16 |
| *D*(~1) *g_0_*(~1) σ(~1) (Isotropic) | 3 | 879.97 | 19.44 | 0.00 | –435.54 |
| *D*(~1) *g_0_*(~bk + Sex) σ(~1) Φ(~1) | 7 | 916.35 | 55.82 | 0.00 | –448.74 |
| *D*(~1) *g_0_*(~bk) σ(~Sex) Φ(~1) | 7 | 916.81 | 56.28 | 0.00 | –448.97 |
| *D*(~1) *g_0_*(~bk + Sex) σ(~Sex) Φ(~1) | 7 | 919.62 | 59.09 | 0.00 | –448.53 |
| *D*(~1) *g_0_*(~bk) σ(~Species) Φ(~1) | 7 | 935.80 | 75.27 | 0.00 | –458.46 |
| *D*(~1) *g_0_*(~bk + Species) σ(~Species) Φ(~1) | 9 | 939.74 | 79.21 | 0.00 | –456.58 |
| *D*(~1) *g_0_*(~bk + Species) σ(~1) Φ(~1) | 7 | 945.41 | 84.89 | 0.00 | –463.27 |

^a^ Number of model parameters.

^b^ Akaike’s Information Criterion corrected for small sample size.

^c^ Relative difference between AIC*_c_* of model and the highest ranked model.

^d^ Model weight.

^e^ log-likelihood of model.

**Fig. B1** Two-dimensional detection probabilities as functions of distance away from the activity center of an individual (●). Based on parameter estimates from the most parsimonious spatial capture-recapture model with an anisotropic half-normal detection function, and latent two-class finite mixtures modeled on the probability of detection at the activity center and the spatial scale of detection


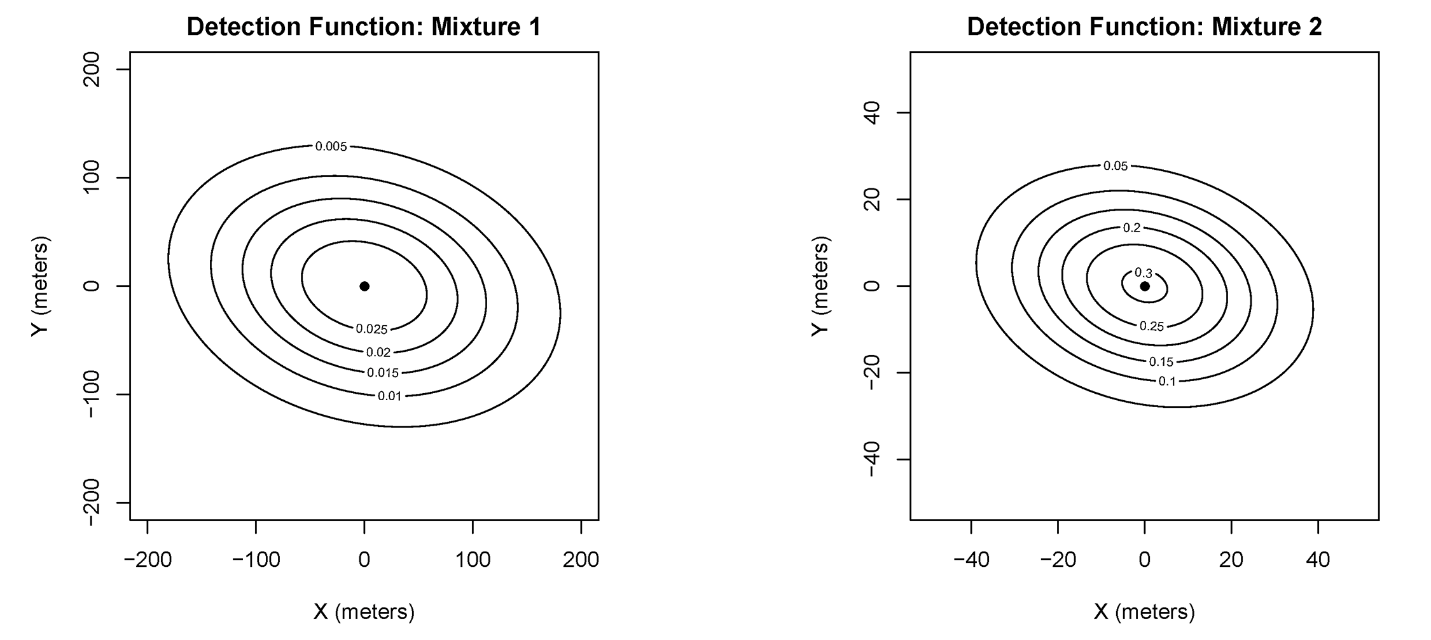

Supplement: S2 Appendix — (DOCX) [file pone.0238870.s002.docx]
